# Supplementary figures and images for: Endometrial microbiota composition is associated with reproductive outcome in infertile patients
Source: Microbiome. 2022 Jan 4;10:1. doi: 10.1186/s40168-021-01184-w (PMC8725275; doi:10.1186/s40168-021-01184-w)

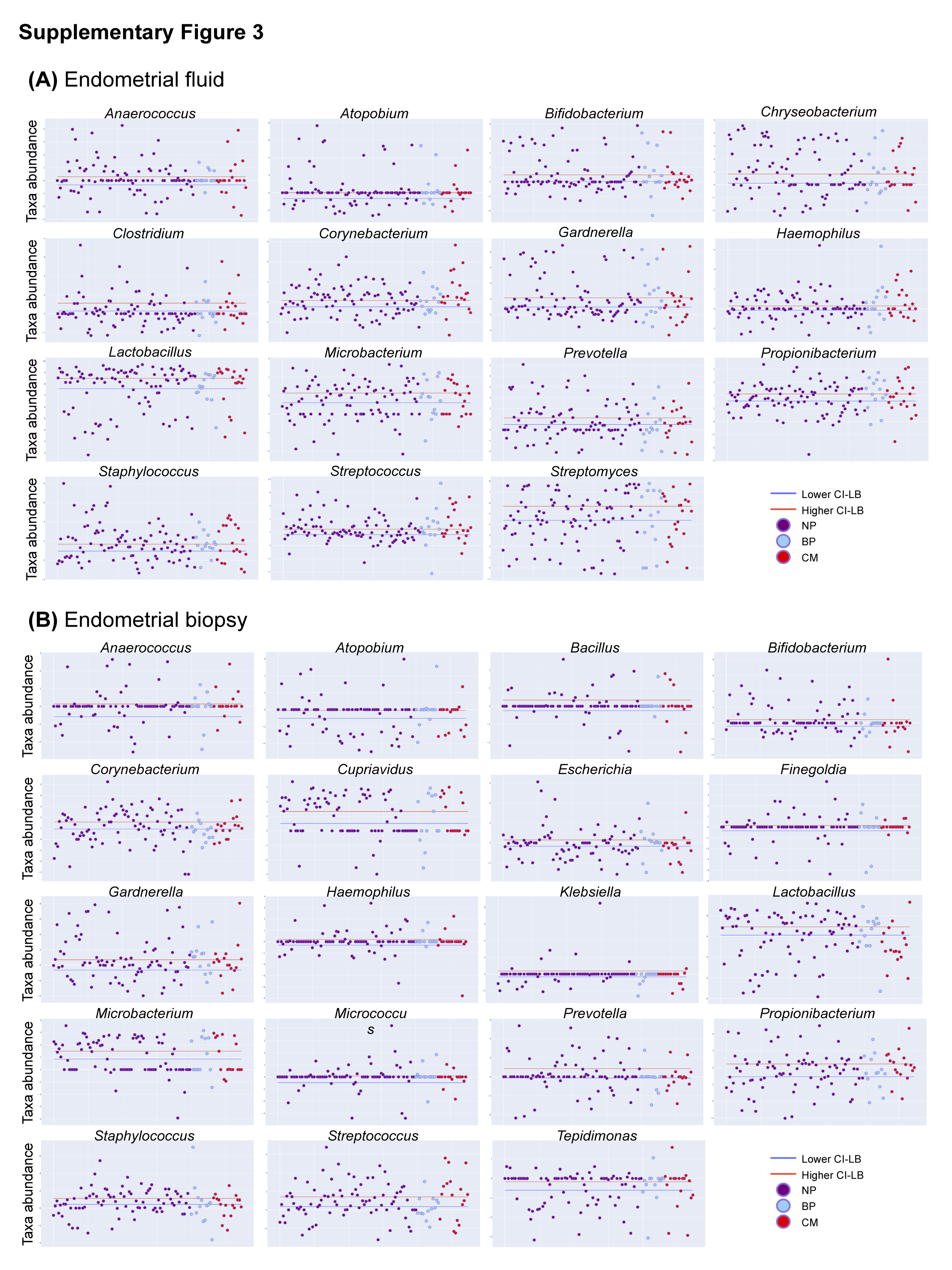

Supplement: Supplementary file 4 — Additional file 3 : Supplementary Figure 3. Confidence intervals and reference ranges of bacterial taxa detected in patients with live birth. The endometrial microbiome from patients with a live birth was analysed in (A) endometrial fluid and (B) endometrial biopsy samples to determine the reference ranges for each evaluated taxon. The confidence interval displays the relative abundance for all assessed patients, revealing the healthy ranges of abundance for the taxa in the tested panel. The healthy distribution was used to define the 95% confidence interval (red line) and taxa abundance in patients with poor reproductive outcomes—no pregnancy, biochemical pregnancy, and clinical miscarriage—were comparatively represented. A taxa abundance of 0 was assigned when the bacteria was not detected in a given sample. BP, biochemical pregnancy; CM, clinical miscarriage; LB, live birth; NP, no pregnancy. [file 40168_2021_1184_MOESM4_ESM.tif]
